# Electron-bunch lengthening on higher-harmonic oscillations in storage-ring free-electron lasers

**DOI:** 10.1107/S160057751700916X

**Published:** 2017-08-07

**Authors:** Norihiro Sei, Hiroshi Ogawa, Shuichi Okuda

**Affiliations:** a Research Institute for Measurement and Analytical Instrumentation, National Institute of Advanced Industrial Science and Technology, 1-1-1 Umezono, Tsukuba, Ibaraki 305-8568, Japan; b Quantum and Radiation Engineering, Osaka Prefecture University, 1-1 Gakuen-cho, Sakai, Osaka 599-8531, Japan

**Keywords:** free-electron laser, higher-harmonic oscillation, bunch length, detuning curve

## Abstract

The influence of higher-harmonic free-electron laser (FEL) oscillations on an electron beam have been studied by measuring its bunch length at the NIJI-IV storage ring. The bunch length and the lifetime of the electron beam were measured, and were observed to have become longer owing to harmonic lasing, which is in accord with the increase of the FEL gain. It was demonstrated that the saturated FEL power could be described by the theory of bunch heating, even for the harmonic lasing. Cavity-length detuning curves were measured for the harmonic lasing, and it was found that the width of the detuning curve was proportional to a parameter that depended on the bunch length. These experimental results will be useful for developing compact resonator-type FELs by using higher harmonics in the extreme-ultraviolet and the X-ray regions.

## Abstract 2

The influence of higher-harmonic free-electron laser oscillations on an electron beam have been clarified by measuring the bunch length of the electron beam.

## 1. Introduction   

The generation of higher-harmonic oscillation is an effective technique for shortening the wavelength of a free-electron laser (FEL) oscillation. Because this technique requires an optical cavity, the working region of the wavelength of the higher-harmonic oscillation is limited by the performance of the optical cavity. However, pioneering studies on higher-harmonic FEL oscillations have made significant advances since the dawn of the development of FEL devices (Colson, 1981 ▸; Jerby & Gover, 1986 ▸). A third-harmonic FEL oscillation was achieved for the first time with a linear accelerator during the 1980s (Benson & Madey, 1989 ▸), and over the years several groups have achieved FEL oscillations on seventh or lower harmonics (Warren *et al.*, 1990 ▸; Kato *et al.*, 1998 ▸; Neil *et al.*, 2001 ▸; Hajima *et al.*, 2001 ▸; Wu *et al.*, 2008 ▸; Sei *et al.*, 2012*a* ▸). Recently, the technique of higher-harmonic oscillation has been examined for its application in an X-ray FEL oscillator (Dai *et al.*, 2012 ▸), which is based on an advanced energy-recovery linear accelerator and is equipped with a high-reflectivity crystal in the X-ray cavity (Kim *et al.*, 2008 ▸). By utilizing this technique, the FEL oscillation in the X-ray region can be realised using an electron beam with a relatively low energy of 3.5 GeV (Deng & Dai, 2013 ▸).

Previously, we have carried out advanced studies to determine the characteristics of higher-harmonic FEL oscillations using the infrared FEL system of the NIJI-IV storage ring (Yamazaki *et al.*, 1998 ▸; Sei *et al.*, 2009 ▸). We demonstrated the generation of FEL oscillations using up to the seventh harmonic (Sei *et al.*, 2012*b* ▸), which is the highest harmonic order ever achieved in the field of higher-harmonic FEL oscillations. In addition, it was demonstrated that the higher harmonic was useful for shortening the FEL wavelength using the higher interference of the dielectric multilayer mirrors in the optical cavity (Sei *et al.*, 2012*c* ▸). Several aspects of the FEL gain for the higher-harmonic FEL oscillations were clarified in detail by the experiments performed with the NIJI-IV infrared FEL system (Sei *et al.*, 2010 ▸, 2014*b* ▸).

However, systematic studies on the influence of higher-harmonic FEL oscillations on the electron beam have not been reported. It is important for the development of the beam physics to investigate the behaviour of the electron beam on the higher-harmonic FEL oscillation. The storage-ring FEL oscillation causes bunch lengthening of the electron beam *via* energy spreading (Renieri, 1979 ▸). Thus, we observed the bunch length of the electron beam on higher-harmonic FEL oscillations by direct and indirect methods. It was clarified that the behaviour of the electron beam on higher-harmonic FEL oscillation could be described using the theory of bunch heating in the storage ring (Elleaume, 1984*a* ▸). In this article we report on a variety of such observational results of the electron beam on the higher-harmonic FEL oscillation using the NIJI-IV infrared FEL system.

## 2. Observation of bunch lengthening with a dual streak camera   

The FEL receives energy from the electron beam while interacting with it and causes a spread in the energy of the electron beam. In the case of a storage-ring FEL, the relative energy spread σ_γ_/*γ* has the following relationship with the bunch length of the electron beam σ_l_ (Wiedemann, 1995 ▸),
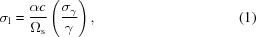
where *c* is the speed of light, α is the momentum compaction factor, and Ω_s_ is the angular synchrotron frequency. As is evident from equation (1), an increase in the energy spread due to the FEL oscillation leads to an increase in the bunch length (Roux *et al.*, 1997 ▸). Because the FEL gain is inversely proportional to the bunch length, the bunch lengthening suppresses the amount of FEL oscillation. Furthermore, an increase in the energy spread causes an increase in the electron-beam size at positions in the storage ring that have a large dispersion function. However, the dispersion function is almost zero along the two long straight sections, where each optical klystron is installed in the NIJI-IV (Sei *et al.*, 2003 ▸). For these two regions, the FEL gain is not affected by an increase in the beam size on the FEL oscillation. Therefore, the saturation of the FEL power is caused by an increase of the energy spread and the bunch length of the FEL systems in the NIJI-IV. When the power of the storage-ring FEL saturates, the FEL gain on the *n*th harmonic *G*_*n*_ is equal to the loss of the optical cavity *l*_c_. According to the one-dimensional FEL theory on the bunch heating process using the optical klystron (Elleaume, 1984*a* ▸), the following approximation for the bunch length holds true (Sei *et al.*, 2003 ▸),
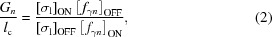
where the suffixes ‘ON’ and ‘OFF’ denote the state of the FEL oscillation (Billardon *et al.*, 1985 ▸); namely, [σ_l_]_ON_ and [σ_l_]_OFF_ are the laser-on and laser-off bunch lengths, respectively. The modulation factor for the *n*th harmonic *f*_γ*n*_ is given by
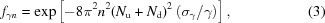
where *N*_u_ is the number of periods in one undulator section of the optical klystron and *N*_d_ is the number of periods of the fundamental wavelength passing over an electron in the dispersive section (Elleaume, 1984*b* ▸). By using equation (1), equation (3) can be described using the bunch length in substitution for the energy spread. It is possible to quantitatively evaluate the influence of the FEL oscillation on the electron beam by observing the influence of the bunch length on the FEL oscillation.

**Figure 1:**
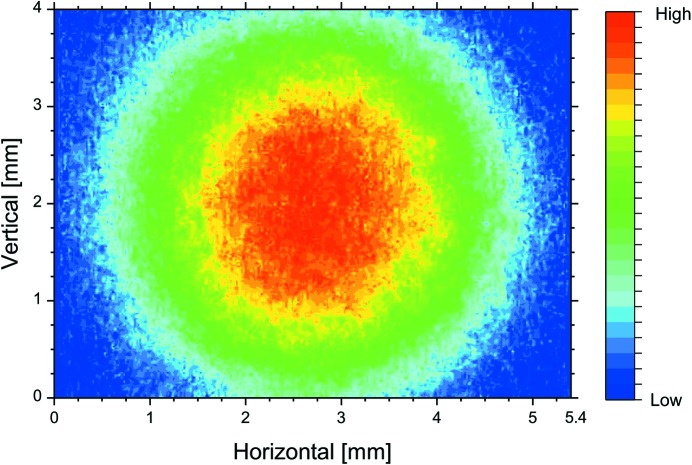
Third-harmonic FEL profile measured using a charge-coupled device camera at a position 0.8 m away from the downstream cavity mirror. The spot size at the observed position was 2.55 mm, which is consistent with the ideal spot size of 2.40 mm.

**Figure 2:**
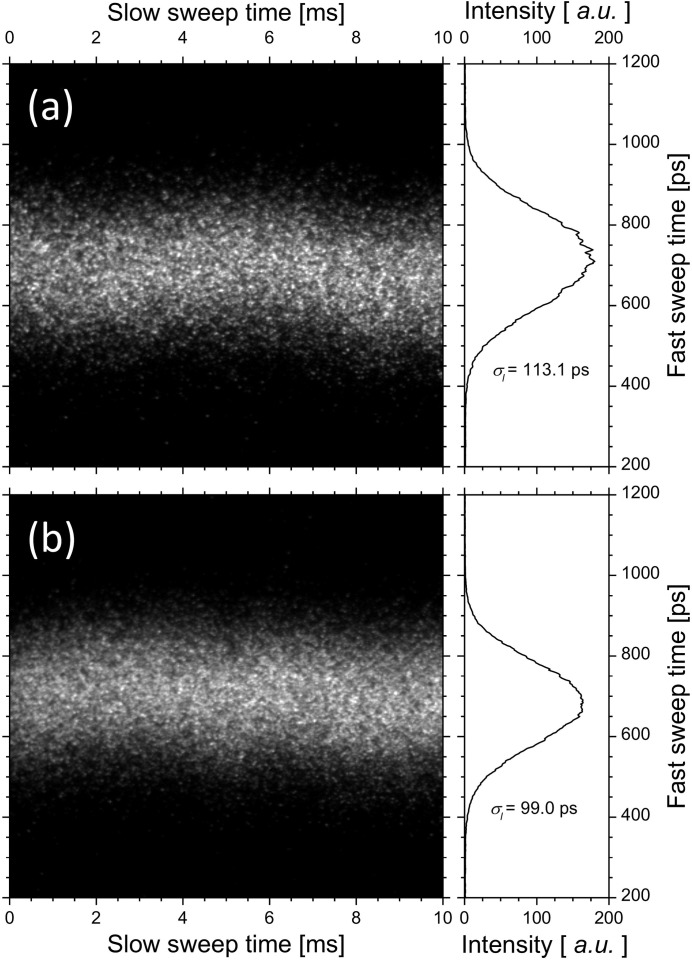
Bunch lengths measured with a dual-sweep streak camera on (*a*) the fundamental FEL oscillation and (*b*) the third-harmonic FEL oscillation.

**Figure 3:**
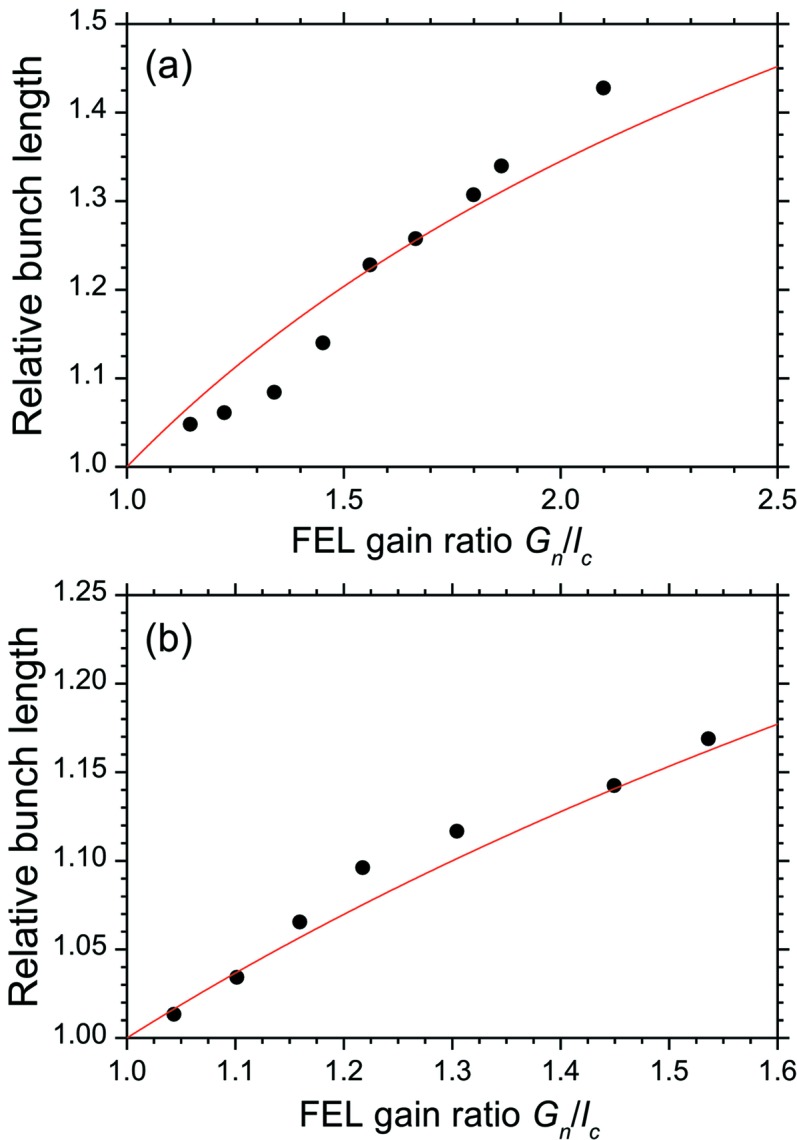
Measured values of the dependence of the relative bunch length on the *G*_*n*_/*l*_c_ ratio on (*a*) the fundamental FEL oscillation and (*b*) the third-harmonic FEL oscillation. The solid curves are the calculated dependence values obtained using equation (2).

Then, we observed the synchrotron radiation from a bending magnet with a dual-sweep streak camera of 2 ps resolution and measured the bunch lengths on the fundamental and third-harmonic FEL oscillations (Sei *et al.*, 2008 ▸). The electron energy in the infrared FEL experiments operated in single electron-bunch mode was 310 MeV. The relative energy spread of the electron bunch was 4.0 × 10^−4^ in the single-bunch mode, and the bunch length, which was independent of the electron-beam current below 5 mA, was approximately 90 ps (Sei *et al.*, 2009 ▸). Dielectric multilayer mirrors having target wavelengths of 870 nm were used in the experiments (Sei *et al.*, 2010 ▸). As shown in Fig. 1 ▸, the higher-harmonic FEL whose profile was almost a perfect TEM_00_ mode was obtained in the NIJI-IV infrared FEL system. Table 1 ▸ shows the parameters of the optical klystron ETLOK-III and the values of the saturated FEL power derived from the higher-harmonic FEL experiments. The deflection parameter of the ETLOK-III (Sei *et al.*, 2002 ▸), *K*, was 2.09 and 4.14 for the fundamental and the third-harmonic FELs, respectively, and their corresponding values of threshold current were 2.3 and 3.5 mA. As shown in Fig. 2 ▸, the bunch lengths on the fundamental and third-harmonic FEL oscillations were different for the same value of single-bunch current. Because the FEL gain for the fundamental FEL is higher compared with that for the third-harmonic FEL, the difference in bunch length with respect to the order of the higher harmonic should be based on investigations of the dependence of the bunch length on the ratio *G*_*n*_/*l*_c_, as opposed to the beam current. Fig. 3 ▸ shows the measured dependence of the relative bunch length on the ratio in the fundamental and third-harmonic FEL oscillations. The solid curves in Fig. 3 ▸, which are the calculated dependence using equation (2), are roughly in accord with the measured data. Thus, the bunch lengthening on the higher-harmonic FEL oscillation can be also described by equation (2), which is based on the theory of bunch heating for the fundamental FEL oscillation. We demonstrate that the theory of bunch heating is applicable to the higher-harmonic FEL oscillations.

## 3. Saturated power of the storage-ring FEL   

**Figure 4:**
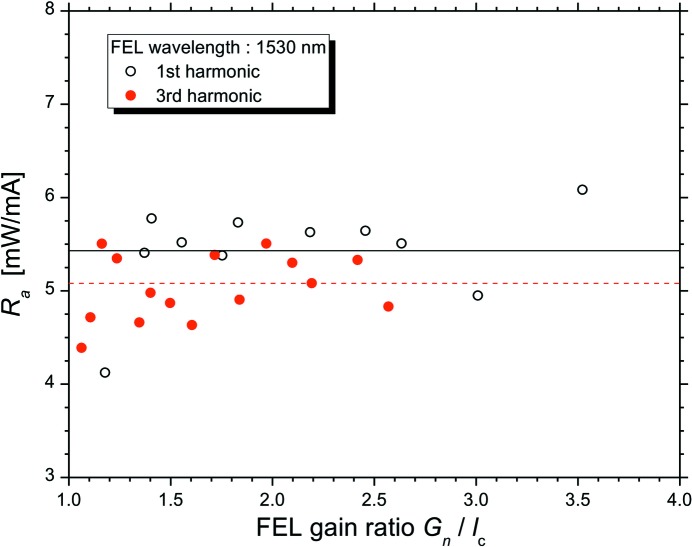
Dependence of the measured ratio *R*_a_ on the FEL gain ratio *G*_*n*_/*l*_c_ in the fundamental FEL oscillation (open circle) and the third-harmonic FEL oscillation (solid circle). The solid and dotted lines are the average values for the fundamental FEL oscillation and the third-harmonic FEL oscillation, respectively, in the region *G*_*n*_/*l*_c_ > 1.1.

An electron beam serves as the laser medium for the FEL interaction, and a FEL oscillation reaches saturation when more energy cannot be extracted from the electron beam. According to the theory of bunch heating extended for the higher-harmonic FEL oscillations, the saturated output power of the storage-ring FEL, *P*_T_, has the following relationship with the ratio *G*_*n*_/*l*_c_ (Elleaume *et al.*, 1984 ▸; Sei *et al.*, 2014*a* ▸),
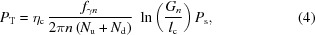
where η_c_ is the optical cavity efficiency, which is defined as the quotient of the output mirror transmission divided by the cavity loss. The symbol *P*_s_ is the total synchrotron radiation power emitted in the entire storage ring, and it is proportional to the electron-beam current *I*_b_. This equation indicates that *P*_T_ is a function of the ratio *G*_*n*_/*l*_c_, and, subsequently, also of the bunch length on the FEL oscillation. To experimentally confirm that equation (4) holds for any arbitrary order of higher harmonic, we measured the ratio *R*_a_ as follows,
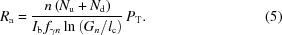
The saturated output power was measured using a calibrated power meter (Coherent Inc., OP-2 IR) with the fundamental and the third-harmonic FEL oscillations at a wavelength of ∼1530 nm (Sei *et al.*, 2017 ▸). Fig. 4 ▸ depicts a plot of the relationship between the measured values of *R*_a_ and the *G*_*n*_/*l*_c_ ratio. It is noted that the measured values of *R*_a_ were roughly constant for both the harmonics in the large area of *G*_*n*_/*l*_c_. The mean values of the measured *R*_a_ were 5.4 ± 0.52 for the fundamental FEL oscillation and 5.1 ± 0.32 for the third-harmonic FEL oscillation, and it was confirmed that they were almost equal. Therefore, the theory of bunch heating is valid for the higher-harmonic oscillation in the storage-ring FEL, and the saturated power of the storage-ring FEL can be described using the effect of bunch lengthening on the higher-harmonic oscillation.

## 4. Increase of electron-beam lifetime in higher-harmonic FEL oscillations   

Generally, the bunch lengthening increases the lifetime of the electron beam in the storage ring. It has been previously reported that the lifetime was increased by the bunch lengthening on the FEL oscillation. For a low-energy storage ring such as the NIJI-IV, the Touschek effect mainly influences the lifetime. The Touschek lifetime *τ*_T_ can be represented using the momentum acceptance (Δ*p*/*p*)_c_ by the following equation (Wallén, 2003 ▸),
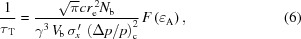
where γ, *r*_e_, *N*_b_ and 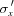 are the energy of the electron expressed in units of its rest energy, the classical electron radius, the number of electrons in a bunch, and the angular divergence of the electron beam in the horizontal plane, respectively. The symbol *V*_b_ denotes the volume of the bunch, and it is inversely proportional to the bunch length. The parameter *∊*_A_ is given by
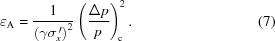
In the case of ∊_A_ << 1, the function *F*(∊_A_) is approximately given by the following equation,
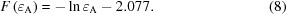
In the infrared experiments, the horizontal and vertical betatron tunes of the storage-ring NIJI-IV were 2.29 and 1.38, respectively. The horizontal and vertical beam sizes at the centre of the long straight section were measured to be 0.9 and 0.2 mm at a beam current of 3 mA, respectively. The beam sizes were almost constant in the 2–5 mA current region. Although the dispersive function was almost zero in the two long straight sections, it increased in the sections containing the bending magnets (Sei *et al.*, 2003 ▸). Subsequently, the horizontal beam size and the horizontal angular divergence increased due to energy spreading on the FEL oscillation. The lifetime was also increased owing to other factors besides the bunch lengthening.

**Figure 5:**
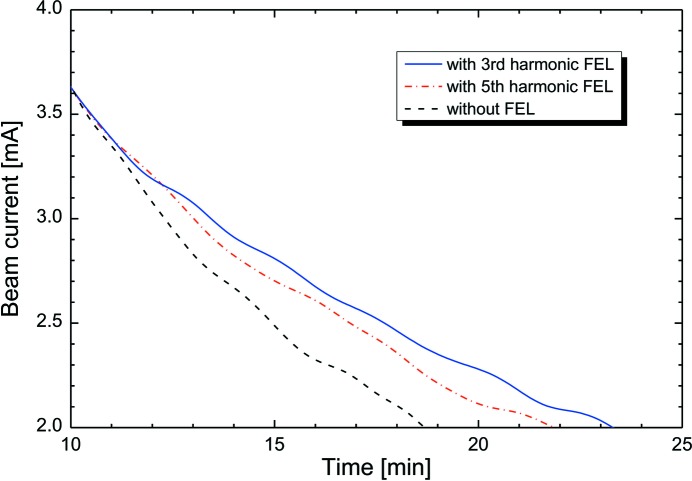
Measured decay curves of the electron-beam current in the presence of the third-harmonic FEL oscillation (solid line) and the fifth-harmonic FEL oscillation (chain line) and in the absence of an FEL oscillation (dotted line). The FEL wavelengths were 1530 nm for third-harmonic lasing and 890 nm for fifth-harmonic lasing.

To investigate the influence of the order of the higher-harmonic FEL oscillation on the lifetime, we measured the electron beam current on the fifth-harmonic FEL oscillation at the wavelength 890 nm and on the third-harmonic FEL oscillation at the wavelength 1530 nm. As shown in Table 1 ▸, the two FEL oscillations could be operated under almost the same conditions as for the *K* and *N*_d_ values. In other words, the electron beam orbit in the third-harmonic FEL experiment was the same as that in the fifth-harmonic FEL experiment. The threshold current in the NIJI-IV infrared FEL system for the third-harmonic FEL oscillation was almost equal to that of its fifth-harmonic. Furthermore, it was possible to observe the influence of the order of the higher-harmonic FEL oscillation on the electron beam at a certain *G*_*n*_/*l*_c_ ratio. Fig. 5 ▸ shows the decay curves of the electron-beam currents in the presence or absence of the higher-harmonic FEL oscillations. Because the electron beam orbit was set on the central axis of the ETLOK-III, the closed orbit distortion of the electron beam in NIJI-IV increased in the infrared FEL experiments. When the *K* value was 5.6, the momentum acceptance was approximately 1.5 × 10^−3^. As shown in Fig. 5 ▸, the Touschek lifetime was only 9 min at a electron-beam current of 3.0 mA in the single-bunch operation. It can be deduced that the lifetime of the electron beam increased by oscillating the FEL and its length increased for decreasing order of the higher harmonics. Table 2 ▸ shows the values of the products of the current and lifetime in the presence and absence of the higher-harmonic FEL oscillations for a current region of 3.6–3.0 mA. Generally, the product at a storage ring was constant despite the differences in the values of current (Bernardini *et al.*, 1963 ▸). The products for the third and the fifth harmonics were, respectively, 1.53 ± 0.22 times and 1.34 ± 0.11 times higher than that for the case where the FEL oscillation was absent. Table 2 ▸ also shows the products calculated with the electron-beam parameters evaluated by the bunch lengthening on the higher-harmonic FEL oscillations at a current of 3.6 mA. According to the estimation, the calculated products for the third and the fifth harmonics were, respectively, 1.52 times and 1.25 times higher than the case where the FEL oscillation was absent. These are in accord with the measured values. These experimental results suggest that the theory of bunch heating can describe the properties of the electron beam on the higher-harmonic FEL oscillations.

## 5. Detuning of the cavity length   

It is known that a macrotemporal structure appears in the storage-ring FEL due to a detuning of the optical cavity (Elleaume *et al.*, 1984 ▸; Couprie *et al.*, 1993 ▸; Roux *et al.*, 1997 ▸; Litvinenko *et al.*, 2001 ▸; Sei *et al.*, 2004 ▸). The minute detuning causes period oscillations in the FEL output power and the bunch length, whose typical time scales are from 1 ms to 100 ms. Such period structures were observed on the higher-harmonic FEL oscillations in the NIJI-IV infrared FEL system. However, we are interested in the longer-term behaviour of the FEL output power and the bunch length on the higher-harmonic FEL oscillations. In this paper, we consider time-averaged physical quantities of millisecond order.

**Figure 6:**
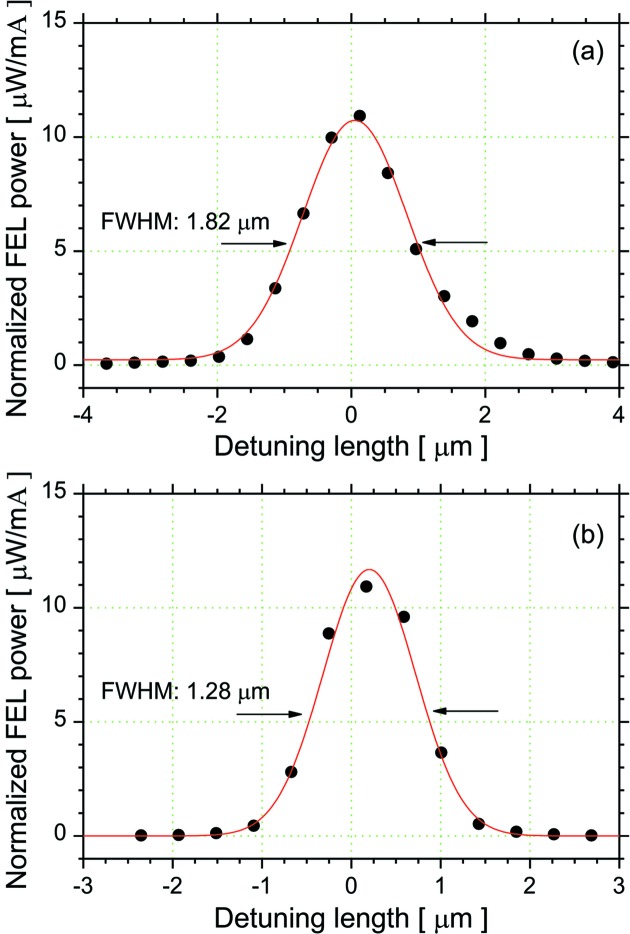
Cavity-length detuning curves for third-harmonic FEL oscillation (*a*) and fifth-harmonic FEL oscillation (*b*) at a FEL gain ratio *G*_*n*_/*l*_c_ of 2.2. The solid lines are the Gaussian fitting curves of the measured data.

Since the FEL gain is proportional to the electron density in the electron bunch, the FEL gain has a Gaussian distribution with a standard deviation equal to the bunch length in the direction of the electron orbit. When the cavity-length detuning is much smaller than the bunch length, the FEL can reach an oscillation even for a low *G*_*n*_/*l*_c_ ratio. The bunch length is an essential parameter for the cavity-length detuning curve, which describes a relationship between the detuning length and the FEL intensity. When the maximum FEL gain is fixed, it is anticipated that the FEL intensity at a certain detuning length should be higher as the bunch length is longer. To confirm this estimate, we measured the cavity-length detuning curves on the fifth-harmonic FEL oscillation at 890 nm and the third-harmonic FEL oscillation at 1530 nm. As mentioned in the last section, these higher-harmonic FELs could oscillate under the same conditions of magnetic field as the ETLOK-III, and the threshold electron-beam currents of these higher-harmonic FELs were approximately equal. Fig. 6 ▸ shows the cavity-length detuning curves on two higher-harmonic FEL oscillations at a *G*_*n*_/*l*_c_ ratio of 2.2. The bunch lengths calculated using equation (2) for the third-harmonic FEL oscillation and the fifth-harmonic FEL oscillation are 122 and 107 ps, respectively, at a *G*_*n*_/*l*_c_ ratio of 2.2. On the other hand, the full width at half-maximums of the detuning curves, Δ*D*, were measured to be 1.89 µm on the third-harmonic FEL oscillation and 1.28 µm on the fifth-harmonic FEL oscillation. These experimental results demonstrated for the first time that the values of Δ*D* were different due the difference in the bunch lengths on the FEL oscillations, despite the conditions of the insertion device and the *G*_*n*_/*l*_c_ ratio being the same. We could realise such comparisons using the higher-harmonic FEL oscillations.

**Figure 7:**
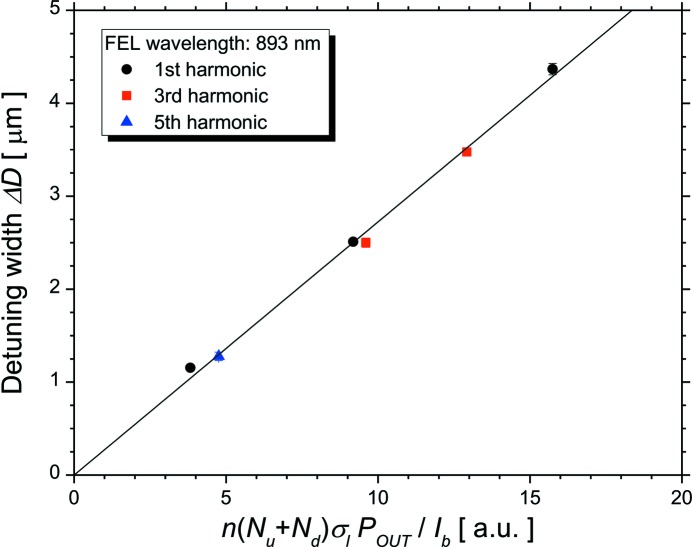
Correlation between *n*(*N*_u_ + *N*_d_)σ_l_ *P*_OUT_/*I*_b_ depending on the bunch length and the width of the cavity-length detuning curve Δ*D*. The circle, square and triangle symbols are data points for fundamental, third-harmonic and fifth-harmonic lasing, respectively. The solid line is a fitting line with collinear approximation.

We experimentally investigate a relationship between the width of the detuning curve and the bunch length on the higher-harmonic FEL oscillation. The detuning can be understood as a gap between the electron bunch and the resonated light pulse in the direction of the electron-beam orbit. When a gap exists in the optical cavity, the number of FEL interactions for the light pulse is proportional to the bunch length. The electron bunch has a delay with respect to the light pulse due to its undulating motion in the optical klystron, which is proportional to *n*(*N*_u_ + *N*_d_) (Deacon *et al.*, 1984 ▸). This delay increases the region of the FEL interaction linearly, and is considered to have a positive correlation with Δ*D*. Moreover, the detuning curve represents the amplification factor of the light pulse caused by the FEL interaction, such that Δ*D* would be proportional to the FEL intensity normalized by the electron-beam current, *P*_T_/*I*_b_. When Δ*D* is much smaller than the bunch length, it is expected that Δ*D* is approximately proportional to the product of these parameters [σ_l_, *n*(*N*_u_ + *N*_d_) and *P*_T_/*I*_b_], despite the FEL interaction being a nonlinear phenomenon between the electron bunch and the light pulse. Fig. 7 ▸ plots the correlation between the product of *n*(*N*_u_ + *N*_d_)σ_l_ *P*_OUT_/*I*_b_ and Δ*D* in the higher-harmonic FEL experiments. The Pearson correlation coefficient between these parameters was evaluated to be 0.9996 for all the higher harmonics. It is noted that the width of the detuning curve was perfectly proportional to the product *n*(*N*_u_ + *N*_d_)σ_l_ *P*_OUT_/*I*_b_. Because the FEL intensity is a function of the bunch length given by equations (2) and (4), Δ*D* can be directly described in terms of the bunch length. An expression for the width of the detuning curve was determined experimentally, which could be applied to higher-harmonic FEL oscillations.

## 6. Conclusion   

We developed higher-harmonic FELs using the infrared FEL system in the NIJI-IV storage ring and observed various phenomena caused by the electron bunch on the higher-harmonic FEL oscillations. The temporal structure of the electron-beam micropulse was measured by a dual-sweep streak camera, and it was confirmed that bunch lengthening occurred on the higher-harmonic FEL oscillations. The saturated power of the storage-ring FEL was also measured on the higher-harmonic FEL oscillation, and it was demonstrated that the power could be described by the theory of bunch heating. It was observed that the third- and the fifth-harmonic FELs could oscillate for the same conditions of the electron beam and optical klystron by changing the optical cavity mirrors. By adjusting the threshold currents for two higher-harmonic FELs to the same value, we could set a condition of the bunch lengths being different for the higher-harmonic FELs despite having the same *G*_*n*_/*l*_c_ ratio. The measured lifetimes of the electron beam on the higher-harmonic FELs were affected by the phenomenom of energy spreading. Moreover, we demonstrated that the full width at half-maximum of the detuning curve was proportional to the product depending on the bunch length in the higher-harmonic FEL oscillation.

The generation of the higher-harmonic oscillation is a technique that allows systems of an extreme-ultraviolet (EUV) FEL and X-ray FEL oscillators (XFELO) to have compact designs. It would be possible to develop a compact EUV FEL system using a higher-harmonic resonator in an optical cavity as a seed light. There are few papers that report on the experimental results of the characteristics of the higher-harmonic FEL; however, there are hardly any reports on the influence of the higher-harmonic FEL oscillation on the electron beam. We clarified this aspect by measuring the bunch length on the harmonic lasing in the NIJI-IV infrared FEL system. We believe that these experimental results will be useful for developing compact EUV FEL and XFELO systems.

## Figures and Tables

**Table 1:** Parameters of the optical klystron ETLOK-III and saturated FEL power in the higher-harmonic FEL experiments

Wavelength (nm)	Cavity loss	Harmonic order	*K* value	*n*(*N*_u_ + *N*_d_)	Threshold current (mA)	*P*_out_ at 5 mA (µW)
870	1.0 × 10^−3^	Fundamental	2.09	197	2.3	25
		Third harmonic	4.14	265	3.5	4.4
890	4.5 × 10^−4^	Fundamental	2.13	195	0.55	320
		Third harmonic	4.19	269	0.90	200
		Fifth harmonic	5.52	357	1.5	66
1530	1.2 × 10^−3^	Fundamental	3.03	122	1.0	310
		Third harmonic	5.58	209	1.6	79

**Table 2:** Measured and calculated values of the products of the current and lifetime in the presence and absence of the higher-harmonic FEL oscillations

	Calculated rate of increase of electron-beam lifetime				
Condition	Component of bunch length	Component of beam size and divergence	Total	Measured product of lifetime and current (min mA)	Measured rate of increase of lifetime
FEL off	1	1	1	39.1 ± 2.4	1
Third-harmonic lasing	1.37	1.11	1.52	59.7 ± 7.9	1.53 ± 0.22
Fifth-harmonic lasing	1.19	1.05	1.25	53.6 ± 3.1	1.34 ± 0.11

## References

[bb1] Benson, S. V. & Madey, J. M. J. (1989). *Phys. Rev. A*, **39**, 1579–1581. 10.1103/physreva.39.1579 9901407

[bb2] Bernardini, C., Corazza, G. F., Di Giugno, G., Ghigo, G., Haissinski, J., Marin, P., Querzoli, R. & Touschek, B. (1963). *Phys. Rev. Lett.* **10**, 407–409.

[bb3] Billardon, M., Elleaume, P., Ortega, J. M., Bazin, C., Bergher, M., Velghe, M., Deacon, D. A. & Petroff, Y. (1985). *IEEE J. Quantum Electron.* **21**, 805–823.

[bb4] Colson, W. B. (1981). *IEEE J. Quantum Electron.* **17**, 1417–1427.

[bb5] Couprie, M. E., Litvinienko, V., Garzella, D., Delboulbé, A., Velghe, M. & Billardon, M. (1993). *Nucl. Instrum. Methods Phys. Res. A*, **331**, 37–41.

[bb6] Dai, J., Deng, H. & Dai, Z. (2012). *Phys. Rev. Lett.* **108**, 034502.

[bb7] Deacon, D. A. G., Billardon, M., Elleaume, P., Ortega, J. M., Robinson, K. E., Bazin, C., Bergher, M., Velghe, M., Madey, J. M. J. & Petroff, Y. (1984). *Appl. Phys. B*, **34**, 207–219.

[bb8] Deng, H. X. & Dai, Z. M. (2013). *Chin. Phys. C.* **37**, 102001.

[bb10] Elleaume, P. (1984*a*). *J. Phys. Fr.* **45**, 997–1001.

[bb9] Elleaume, P. (1984*b*). *J. Phys. (Paris)*, **44**(C1), 333–352.

[bb11] Elleaume, P., Ortéga, J. M., Billardon, M., Bazin, C., Bergher, M., Velghe, M., Petroff, Y., Deacon, D. A. G., Robinson, K. E. & Madey, J. M. J. (1984). *J. Phys. Fr.* **45**, 989–996.

[bb12] Hajima, R., Nagai, R., Nishimori, N., Kikuzawa, N. & Minehara, E. J. (2001). *Nucl. Instrum. Methods Phys. Res. A*, **475**, 43–46.

[bb14] Jerby, E. & Gover, A. (1986). *Nucl. Instrum. Methods Phys. Res. A*, **250**, 192–202.

[bb15] Kato, R., Okuda, S., Nakajima, Y., Kondo, G., Iwase, Y., Kobayashi, H., Suemine, S. & Isoyama, G. (1998). *Nucl. Instrum. Methods Phys. Res. A*, **407**, 157–160.

[bb16] Kim, K. J., Shvyd’ko, Y. & Reiche, S. (2008). *Phys. Rev. Lett.* **100**, 244802. 10.1103/PhysRevLett.100.244802 18643591

[bb17] Litvinenko, V. N., Park, S. H., Pinayev, I. V. & Wu, Y. (2001). *Nucl. Instrum. Methods Phys. Res. A*, **475**, 240–246.

[bb18] Neil, G. R., Benson, S. V., Biallas, G., Gubeli, J., Jordan, K., Myers, S. & Shinn, M. D. (2001). *Phys. Rev. Lett.* **87**, 084801. 10.1103/PhysRevLett.87.084801 11497947

[bb19] Renieri, A. (1979). *Nuov. Cim. B*, **53**, 160–178.

[bb20] Roux, R., Couprie, M. E., Hara, T., Bakker, R. J., Visentin, B., Billardon, M. & Roux, J. (1997). *Nucl. Instrum. Methods Phys. Res. A*, **393**, 33–37.

[bb21] Sei, N., Ogawa, H. & Okuda, S. (2017). *J. Appl. Phys.* **121**, 023103.

[bb22] Sei, N., Ogawa, H. & Yamada, K. (2009). *Opt. Lett.* **34**, 1843–1845. 10.1364/ol.34.001843 19529722

[bb23] Sei, N., Ogawa, H. & Yamada, K. (2010). *J. Phys. Soc. Jpn*, **79**, 093501.

[bb24] Sei, N., Ogawa, H. & Yamada, K. (2012*a*). *Appl. Phys. Lett.* **101**, 144101.

[bb25] Sei, N., Ogawa, H. & Yamada, K. (2012*b*). *Opt. Express*, **20**, 308–316. 10.1364/OE.20.000308 22274354

[bb26] Sei, N., Ogawa, H. & Yamada, K. (2012*c*). *J. Phys. Soc. Jpn*, **81**, 093501.

[bb27] Sei, N., Ogawa, H. & Yamada, K. (2014*a*). *JPS Conf. Proc.* **1**, 014005.

[bb28] Sei, N., Ogawa, H., Yamada, K., Koike, M. & Ohgaki, H. (2014*b*). *J. Synchrotron Rad.* **21**, 654–661. 10.1107/S160057751400678X 24971958

[bb29] Sei, N., Ohgaki, H., Mikado, T. & Yamada, K. (2002). *Jpn. J. Appl. Phys.* **41**, 1595–1601.

[bb30] Sei, N., Yamada, K. & Mikado, T. (2004). *Jpn. J. Appl. Phys.* **43**, 577–584.

[bb31] Sei, N., Yamada, K. & Ogawa, H. (2008). *J. Phys. Soc. Jpn*, **77**, 074501.

[bb32] Sei, N., Yamada, K., Ogawa, H., Yasumoto, M. & Mikado, T. (2003). *Jpn. J. Appl. Phys.* **42**, 5848–5858.

[bb33] Wallén, E. (2003). *Nucl. Instrum. Methods Phys. Res. A*, **508**, 487–495.

[bb34] Warren, R. W., Haynes, L. C., Feldman, D. W., Stein, W. E. & Gitomer, S. J. (1990). *Nucl. Instrum. Methods Phys. Res. A*, **296**, 84–88.

[bb13] Wiedemann, H. (1995). *Particle Accelerator Physics II*, ch. 10, p. 350. New York: Springer-Verlag.

[bb35] Wu, Y. K., Benson, S. V., Li, J., Mikhailov, S. F., Neil, G. & Popov, V. (2008). *Proceedings of the 30th International Free Electron Laser Conference*, Gyeongju, Korea.

[bb36] Yamazaki, T., Yamada, K., Sei, N., Ohgaki, H., Sugiyama, S., Suzuki, R., Mikado, T., Noguchi, T., Chiwaki, M., Ohdaira, T. & Toyokawa, H. (1998). *Nucl. Instrum. Methods Phys. Res. B*, **144**, 83–89.

